# Supplementary material for: Genetic bases of resistance to the rice hoja blanca disease deciphered by a quantitative trait locus approach
Source: G3 (Bethesda). 2023 Sep 28;13(12):jkad223. doi: 10.1093/g3journal/jkad223 (PMC10700108; doi:10.1093/g3journal/jkad223)
Supplement: jkad223_Supplementary_Data [file jkad223_supplementary_data.zip › Supplemental_Figure_Legends_G3-2023-404341.docx]

## Supplemental materials

**Figure S1** Segregation distortion in several chromosomes, in the four F_2_ populations. The X-axis represents the position in cM on the chromosome. The Y axis is the frequency of the three F_2_ genotypes: f(A): homozygous female parent (resistant to HBV); f(B): homozygous male parent BBT 50 (susceptible to HBV); f(H): heterozygous. One clearly sees the departure from the expected genotypic frequencies, which in the F_2_ cross are 0.25, 0.25 and 0.5 for f(A), f(B) and f(H), respectively.

**Figure S2** 2-D plot for interaction analysis between genomic regions calculated in the FD2000 x BBT 50 cross. A: RHBV incidence. B: RHBV severity.

**Figure S3** Graphical genotyping showing probable local ancestry for the four RHB-resistant parents (green arrows), in the two QTL regions *qHBV4.1* and *qHBV4.2* on chromosome 4. X-axis: physical coordinates (in bp) along chromosome 4, spanning the region 2.6-4.4 Mbp. Y-axis: each row represents a rice accession. Red: *indica* origin, including the Aus cluster. Blue: *japonica* origin, including temperate, sub-tropical and tropical clusters. Gray: unclear origin. Local ancestry analysis was performed using a set of SNP markers distributed along the whole rice genome and identified previously in 97 diverse rice accessions (Duitama et al. 2015), using custom scripts. Briefly, the accessions were first classified as *indica* or *japonica* by principal component analysis on the SNP set. Then, the most frequent SNP haplotype (MFH) was searched in the *indica* and *japonica* groups using adjacent windows of 224,000 bp (representing ~1 cM in rice). Finally, a simple matching similarity index, SI, was calculated between each haplotype of each accession and the *indica* and *japonica* MFHs. The *indica* or *japonica* origin of the haplotype was attributed if SI > 0.99.
