# Supplementary figures and images for: Genetic bases of resistance to the rice hoja blanca disease deciphered by a quantitative trait locus approach
Source: G3 (Bethesda). 2023 Sep 28;13(12):jkad223. doi: 10.1093/g3journal/jkad223 (PMC10700108; doi:10.1093/g3journal/jkad223)

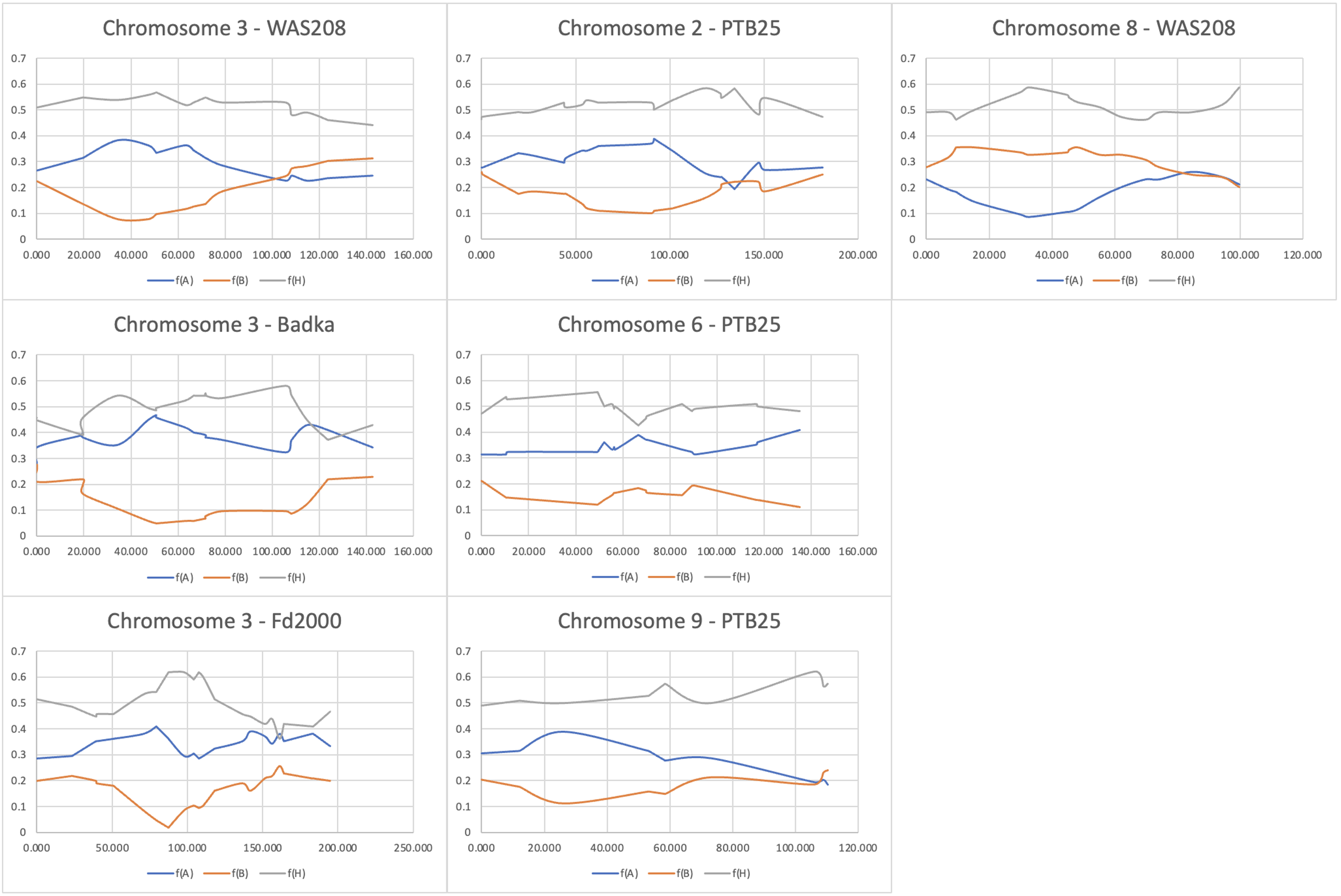

Supplement: jkad223_Supplementary_Data [file jkad223_supplementary_data.zip › Figure_S1_G3-2023-404341.png]

## Slide 1
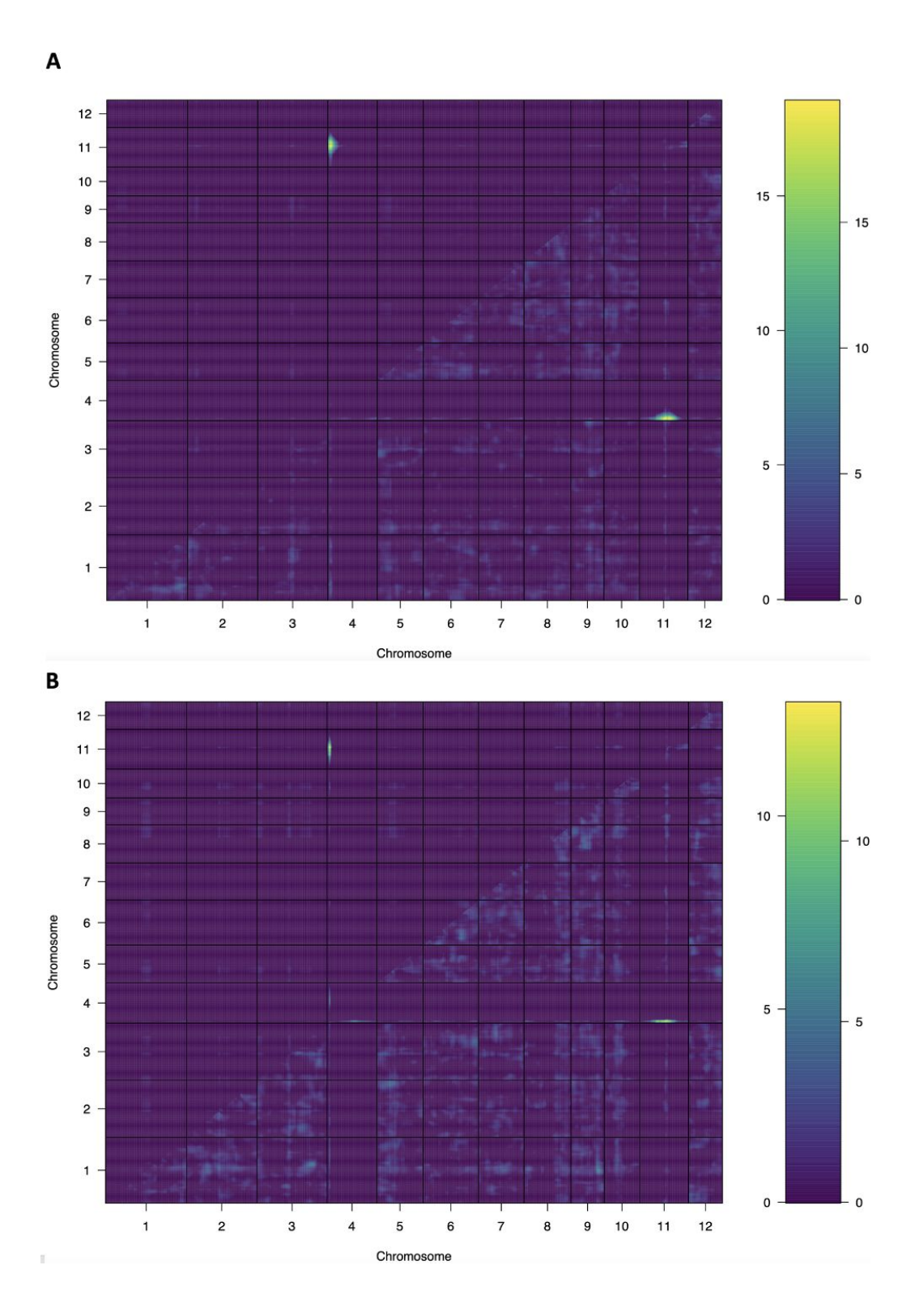

Supplement: jkad223_Supplementary_Data [file jkad223_supplementary_data.zip › Figure_S2_G3-2023-404341.pptx]

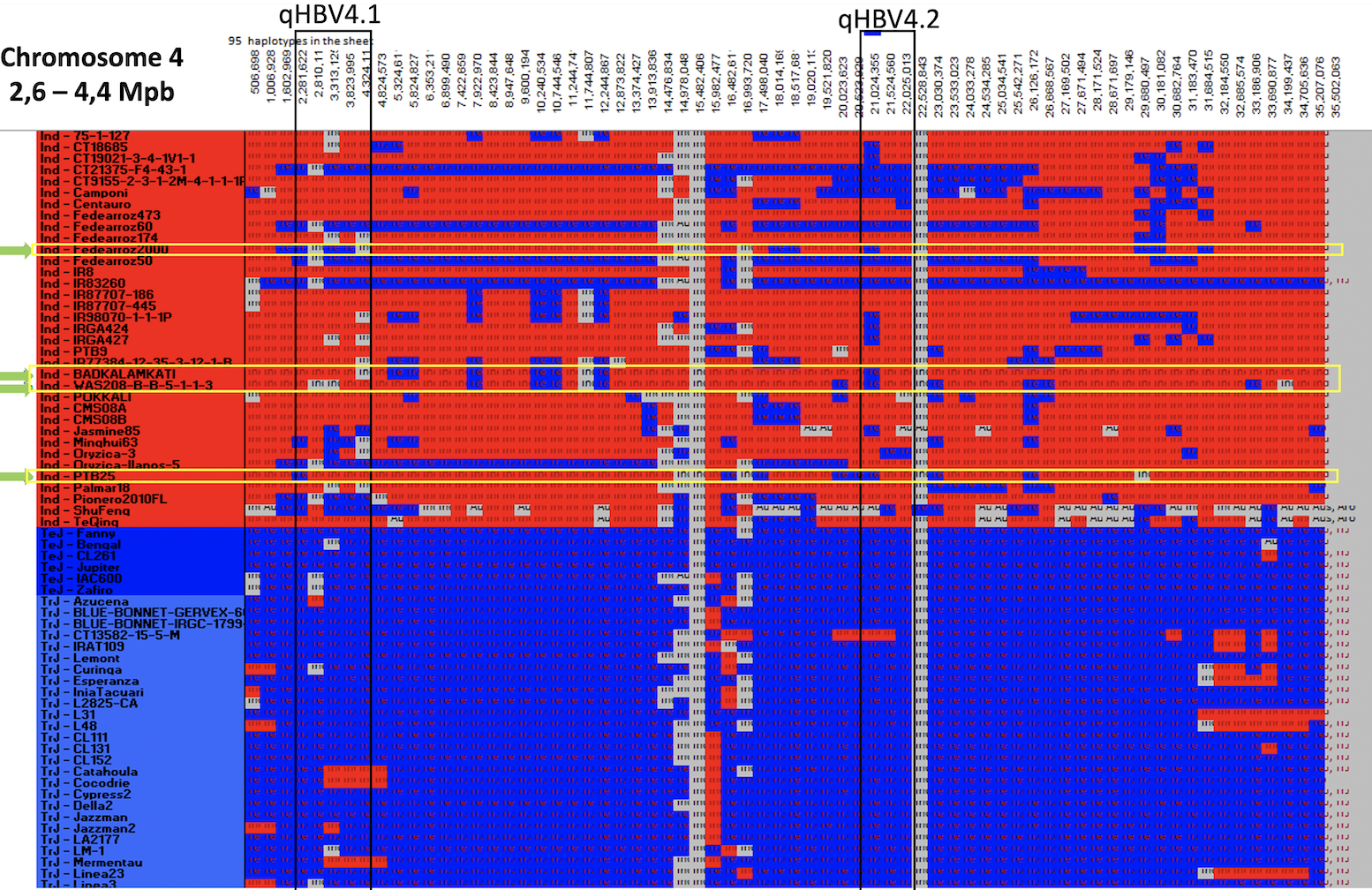

Supplement: jkad223_Supplementary_Data [file jkad223_supplementary_data.zip › Figure_S3_G3-2023-404341.png]
